# Effectiveness of Hospital-Wide Methicillin-Resistant *Staphylococcus aureus* (MRSA) Infection Control Policies Differs by Ward Specialty

**DOI:** 10.1371/journal.pone.0083099

**Published:** 2013-12-10

**Authors:** Rosemarie Sadsad, Vitali Sintchenko, Geoff D. McDonnell, Gwendolyn L. Gilbert

**Affiliations:** 1 Centre for Infectious Diseases and Microbiology – Public Health, Westmead Hospital, Sydney, New South Wales, Australia; 2 Centre for Health Informatics, Australian Institute of Health Innovation, University of New South Wales, Sydney, New South Wales, Australia; 3 Sydney Medical School – Westmead, The University of Sydney, Sydney, New South Wales, Australia; Amphia Ziekenhuis, The Netherlands

## Abstract

Methicillin-resistant *Staphylococcus aureus* (MRSA) is a major cause of preventable nosocomial infections and is endemic in hospitals worldwide. The effectiveness of infection control policies varies significantly across hospital settings. The impact of the hospital context towards the rate of nosocomial MRSA infections and the success of infection control is understudied. We conducted a modelling study to evaluate several infection control policies in surgical, intensive care, and medical ward specialties, each with distinct ward conditions and policies, of a tertiary public hospital in Sydney, Australia. We reconfirm hand hygiene as the most successful policy and find it to be necessary for the success of other policies. Active screening for MRSA, patient isolation in single-bed rooms, and additional staffing were found to be less effective. Across these ward specialties, MRSA transmission risk varied by 13% and reductions in the prevalence and nosocomial incidence rate of MRSA due to infection control policies varied by up to 45%. Different levels of infection control were required to reduce and control nosocomial MRSA infections for each ward specialty. Infection control policies and policy targets should be specific for the ward and context of the hospital. The model we developed is generic and can be calibrated to represent different ward settings and pathogens transmitted between patients indirectly through health care workers. This can aid the timely and cost effective design of synergistic and context specific infection control policies.

## Introduction

Methicillin-resistant *Staphylococcus aureus* (MRSA) is a major cause of preventable nosocomial (or hospital acquired) infections and is endemic in hospitals worldwide [1]. Hospital patients infected with MRSA have a median death rate of 34.2% [2], prolonged lengths of stay [3], [4], and require additional care costing an extra $26,000 USD per episode [4], [5].

Hospitals have applied a wide range of infection control policies to reduce the burden of nosocomial infections. These include contact precautions [6], [7], cohorting by grouping staff with particular patients or allocating staff or patients to designated areas [8], surveillance for multidrug-resistant pathogens [9], decolonisation treatment to reduce carriage of MRSA in patients [8], and restricted or strategic use of antimicrobials to prevent the development of multidrug-resistant infections [10].

The effectiveness of these infection control policies varies significantly across hospital settings, which remains a challenge for evidence-based practice. The prevalence of MRSA amongst *Staphylococcus aureus* isolates collected by health care facilities and hospitals worldwide ranged from less than 1% to greater than 80% [1], [11]. The mixed success of hospital infection control policies internationally has prompted their re-evaluation. Comparative effectiveness studies across different health care contexts are logistically difficult or often infeasible. Mathematical and computational modelling has been successfully applied to evaluate hospital infection control in different settings [12]–[14]. Most studies evaluate infection control for one hospital as a whole or in one specialty ward, such as, an intensive care unit. Several studies demonstrated that disease transmission dynamics simulated for a hospital differs when you explicitly model wards in the hospital [15]–[17]. To our knowledge, no studies to date have evaluated infection control for different ward specialties within one organisation. We conducted a modelling study that compared the differences in the prevalence and nosocomial incidence rate of MRSA, and in the effectiveness of several hospital-wide infection control policies, across three different ward specialties.

## Materials and Methods

### Ethics statement

The use of retrospective, de-identified, patient and hospital administrative data for this study, without patient consent, was approved by the Sydney West Area Health Service Human Research Ethics Committee – Westmead campus (JH/TG HREC2009/12/5.13 (3094) QA), the Sydney South West Area Health Service Human Research Ethics Committee – CRGH Zone (CH62/6/2010-093), and the University of New South Wales Human Research Ethics Advisory Panel (2009-7-67).

### The model

**Figure 1:**
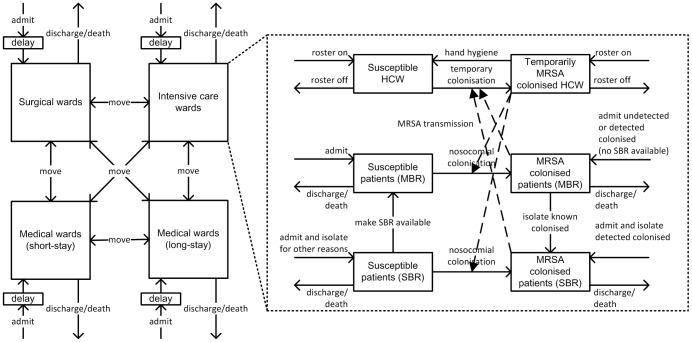
A model of health care worker (HCW) and patient flow dynamics through hospital ward specialties and rooms. Solid arrows represent HCW and patient movement into, out of, and between ward specialties and rooms, and between MRSA susceptible and colonised states. Dashed arrows represent MRSA transmission occurring upon contact between HCWs and patients. When beds in single-bed rooms (SBR) or multiple-bed rooms (MBR) are not available, hospital patient admissions may be delayed and inpatients that are being moved from a ward or room remain in their current bed until the requested beds are made available.

We developed a multiscale simulation model [18]–[20] with AnyLogic® simulation software (www.anylogic.com). We modelled surgical, intensive care, and medical wards, with single- and multiple-bed rooms, of one hospital. Beds in the wards could be opened or closed for a period of time. We employed the System Dynamics modelling method [21] to model health care workers and patients moving into, out of, and between hospital wards and rooms (Figure 1). System Dynamics uses a stock and flow modelling approach. In our model, wards are represented with stocks and health care worker and patient movements are represented with flows. We used Agent Based principles [22], [23] to model MRSA transmission between health care workers, with varying hand hygiene compliance levels, and patients residing in different room types and ward specialties which applied distinct infection control policies.

#### Health care worker and patient flow dynamics

Non-colonised and MRSA colonised patients were admitted to, discharged from, and could move between ward specialties and between single-bed rooms and multiple-bed rooms within wards. The number of patients moving into, out of, and between wards and rooms in wards was based on collected data and did not change when interventions were simulated. The prevalence of MRSA amongst admitted patients reflected the proportion of patients known to be colonised on admission, as informed by patient records. Patient admissions and ward and room movements were delayed when beds were not available. Several wards of the hospital studied (haematology, renal, cardiothoracic surgical, and intensive care wards) routinely screened patients for MRSA on admission to the ward. MRSA colonised patients detected using rapid polymerase chain reaction (PCR) screening tests were isolated in available single-bed rooms on the same day. MRSA colonised patients detected through culture-based tests remained in their current location for two days, and then were isolated in available single-bed rooms. Patients were admitted directly or moved into available single-bed rooms if they were known to be colonised with MRSA or were isolated for reasons other than MRSA. Patients were otherwise allocated to available multiple-bed rooms. When single-bed rooms became available (when previously closed beds were opened or patients were moved from isolation, moved to other wards, or were discharged), known MRSA colonised patients residing in multiple-bed rooms were moved to available single-bed rooms. Non-colonised patients in single-bed rooms could be moved to multiple-bed rooms to make single-bed rooms available. Patients were discharged according to the average length of stay for non-colonised or MRSA colonised patients in that ward specialty. The patient length of stay was modelled with a Poisson distribution. In the hospital studied, most health care workers worked in one ward specialty on a single shift, with nurses making more patient contacts relative to doctors and allied health staff. We modelled nurses that worked in one ward specialty only and serviced both single-bed rooms and multiple-bed rooms. The number of health care workers rostered to a ward reflected the nurse to patient ratio for that ward specialty.

#### MRSA transmission dynamics

Health care workers made contact with different patients daily unless their contacts were cohorted, in which case, they returned to the same patient for each subsequent contact based on a staff contact cohorting probability. Cohorts were reassigned daily due to changing nursing rosters and patient turnover. MRSA transmission between patients through health care workers extends the vector transmission and susceptible-infectious model [24]–[26]. MRSA transmission upon contact depended on the ward specialty, room type, and whether the health care worker performed hand hygiene prior to making contact with patients. Health care workers that acquired MRSA were assumed to be temporarily colonised until adequate hand hygiene was performed. The efficacy of hand hygiene was assumed to be 90% in the baseline case [27], [28]. Patients who acquired MRSA were assumed to remain colonised for the remainder of their hospital admission as colonisation is often longer than the average length of patient stay [29]. Also, decolonisation therapies were found to be marginally effective in removing multisite MRSA carriage, especially in high endemic settings [30].

### Interventions

Parameter variation and sensitivity tests were conducted to evaluate active surveillance for MRSA, ward staffing level, staff contact cohorting, isolation of MRSA patients in single-bed rooms, and staff hand hygiene policies in surgical, intensive care, and medical wards. Each infection control policy was varied over a policy level range of 0% to 100%. For active surveillance, the percentage of all patients admitted to a ward who are tested for MRSA colonisation on admission was varied; ward staffing levels, the health care worker to patient ratio was varied; staff contact cohorting, the probability each health care worker returned to the same patient with each subsequent contact was varied; MRSA patient isolation in single-bed rooms, the percentage of all beds in the ward that are in single-bed rooms was varied; and, staff hand hygiene compliance, the probability health care workers would conduct adequate hand hygiene prior to making contact with patients was varied. The average daily prevalence and incidence rate of MRSA for each ward, over the two-year study period, averaged over 1000 simulations, and their normalised sensitivity to policy variations were measured and compared. The daily prevalence of MRSA was defined as the total number of patients identified with MRSA colonisation or infection, divided by the number of occupied beds on that day. MRSA colonisation was defined by isolation of MRSA from a screening swab or clinical specimen from a patient without clinical signs or symptoms of infection [31]. MRSA infection was defined by isolation of MRSA from a relevant clinical specimen from a patient with clinical evidence of infection. The incidence rate of MRSA was defined as the number of new cases of nosocomial MRSA per 10,000 overnight bed days (OBD). The normalised sensitivity *U_E_(x,y)* for an infinitesimal change in the level of the infection control policy (*x*) and outcome average daily prevalence or incidence rate of MRSA (*y*) at any given point (*x_0_, y_0_*) on the outcome response curve was calculated with the formula [32]:
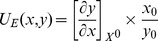



The average daily prevalence and incidence rate of MRSA were sensitive to an infection control policy if the *|normalised sensitivity| > = 1*, that is, there were larger changes in the daily prevalence and incidence rate of MRSA relative to changes in the infection control policy.

### Data

The model was calibrated with data collected from a 980 bed tertiary public hospital in Sydney, Australia, between January 2008 and December 2009 and published data (Table 1). Medical wards were separated into short-stay wards, where the average patient length of stay (ALOS) was ten days or less, and long-stay wards, where the ALOS was greater than ten days. This reflected the bimodal distribution of the ALOS in medical wards identified in the collected data.

**Table 1:** Parameters used in the model.

Model parameter	Value				Source
	Surgical	ICU	Medical (short)	Medical (long)	
Health care worker to patient ratio	1∶8	1∶1	1∶4	1∶6	H
Contacts received per patient daily	5	10	5	5	[34]
Beds in the ward specialty	132	28	59	194	H
Patients admitted daily	33	4	14	35	H
ALOS (days, S)	23	8	9	23	H
ALOS (days, C)	26	10.5	10.5	25.5	H
Patients transferred into the ward daily from other wards (S)	4.6	1.3	2.0	7.0	H
Patients transferred out of the ward daily to other wards (S)	3.9	1.9	6.4	2.5	H
Patients transferred into the ward daily from other wards (C)	0.4	0.2	0.3	0.8	H
Patients transferred out of the ward daily to other wards (C)	0.3	0.3	0.9	0.2	H
Staff hand hygiene compliance (%)	57	64	52	42	H
Hand hygiene efficacy (%)	90	90	90	90	[28]
Patients screened on ward admission (%)	6	100	7	0	H
Patients screened with PCR-test instead of culture-based test (%)	50	50	50	50	A
Delay for culture-based test results (days)	2	2	2	2	H
SBR in the ward	47	25	17	104	H
Patients admitted directly to SBR (%, S)	5	5	5	5	A
Initial patients in MBR (C)	20	1	8	14	H
Initial patients in SBR (C)	11	8	3	17	H
Prevalence of MRSA amongst admitted patients (%)	7	8	9	10	H
Transmission probability in MBR (%)	6	8	5	2	E
Transmission probability in SBR (%)	12	15	11	6	E

A, parameters based on assumptions; ALOS, average patient length of stay; C, MRSA colonised or infected patients; E, parameters estimated from data collected from a tertiary public hospital in Sydney, Australia and subject to sensitivity analysis; H, data collected from a tertiary public hospital in Sydney, Australia; ICU, intensive care units; MBR, multiple-bed rooms; PCR, polymerase chain reaction; S, patients not colonised with MRSA; SBR, single-bed rooms.

MRSA transmission probabilities in single- and multiple-bed rooms in surgical, intensive care, and medical wards were estimated using the OptQuest optimisation tool built into the AnyLogic simulation software package [33]. The values for each parameter were selected from a range of 0%–25%, consistent with the values used in previous studies [28], [34], [35]. The optimisation tool selected combinations of values for the unknown parameters using a scatter search algorithm. A scatter search algorithm is an evolutionary algorithm where an iterative process is used to evaluate parameters against a fitness function, then the best performing parameters are used to seed the selection of the next set of parameters to be evaluated. The algorithm converges to produce a set of parameters that produce a near-optimal or reasonable solution [33], [36]. The combination of eight transmission probabilities that produced a least squares fit between the simulated daily prevalence and incidence rate of MRSA (averaged over 1000 simulations) and that observed in the wards, between April and July 2008, was selected. The MRSA transmission probabilities were estimated to be between 2% to 8% in multiple-bed rooms and between 6% and 15% in single-bed rooms. The higher transmission probabilities estimated for single-bed rooms may reflect more vulnerable patients who were isolated. McBryde et al. [37] observed the average MRSA transmission probability between health care workers and colonised patients in a different Australian hospital to be 17% (95% CI, 9%–25%). Other studies have estimated the MRSA transmission probability in intensive care wards to be between 1.5% and 20% [28], [34], [35]. Sensitivity analyses confirm the simulated prevalence and incidence rate of MRSA, for all ward specialties, to be robust for the estimated MRSA transmission probabilities and to be sensitive to transmission probabilities that were greater than 15% (|normalised sensitivity| > = 1). The daily prevalence of MRSA observed in the wards between April and June 2009 were within the 95% confidence interval of the daily prevalence of MRSA simulated in the wards (averaged over 1000 simulations). For the same three month period, the observed incidence rates of MRSA in long-stay wards (22 cases per 10,000 OBD in surgical wards and 12 cases per 10,000 OBD in long-stay medical wards) were within the 95% confidence interval of the simulated MRSA incidence rates (Table 2). The MRSA incidence rates observed in short-stay wards (83 cases per 10,000 OBD in intensive care wards and 7 cases per 10,000 OBD in short-stay medical wards) were outside of the 95% confidence interval (Table 2). The simulation takes several months to stabilise. Artefacts in the simulated MRSA incidence rate produced in this “burn-in” period may have contributed to these results. This could be improved by calibrating the model with data from six months (instead of three months) prior to the data the model was fitted to.

**Table 2:** The average daily MRSA prevalence and MRSA incidence rate per 10,000 overnight bed days (OBD) in surgical, intensive care (ICU), and medical wards of one hospital, over 2008 and 2009, averaged over 1000 simulation runs.

	Value			
	Surgical	ICU	Medical (short)	Medical (long)
Average daily MRSA prevalence (95% CI)	15.4 (13.0, 17.8)	14.4 (11.0, 17.8)	13.0 (10.8, 15.2)	13.4 (11.8, 15.0)
Average MRSA incidence rate per 10,000 OBD (95% CI)	21.5 (17.2, 25.8)	56.3 (37.9, 74.7)	16.7 (11.3, 22.1)	10.7 (7.6, 13.8)

## Results and Discussion

### Baseline dynamics

**Figure 2:**
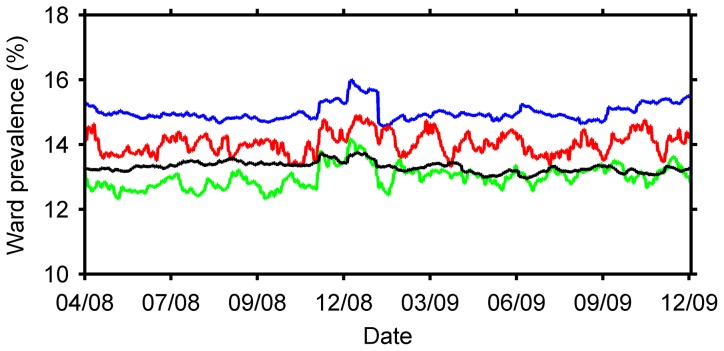
The daily prevalence of MRSA in surgical wards (blue), intensive care wards (red), short-stay medical wards (green), and long-stay medical wards (black) of one hospital, from April 2008 to December 2009, averaged over 1000 simulations.

The average daily prevalence and incidence rate for each ward specialty, under hospital conditions and infection control practices during 2008 and 2009, averaged over 1000 simulation runs, is shown in Table 2. The daily prevalence of MRSA appeared more variable in intensive care (SD of differences, Sd, 0.27) and short-stay medical wards (Sd, 0.12) than in surgical (Sd, 0.09) and long-stay medical wards (Sd, 0.04) (Figure 2). This was likely due to a greater turnover of patients in short-stay wards. Point prevalence surveys should consider the variance in the daily prevalence due to the average length of stay. The increase in the daily prevalence in all ward specialties between November 2008 and February 2009 (Christmas and Southern Hemisphere summer) could be due to bed closures over this period and longer lengths of stay associated with MRSA colonised patients. There are more patients attempting to be admitted to a particular ward than are being discharged or moved out to other wards. An increase in the number of patients admitted directly to a ward would not change the results of this analysis unless the number of patients moved into and out of, and discharged from the wards were also changed.

### Active surveillance

**Figure 3:**
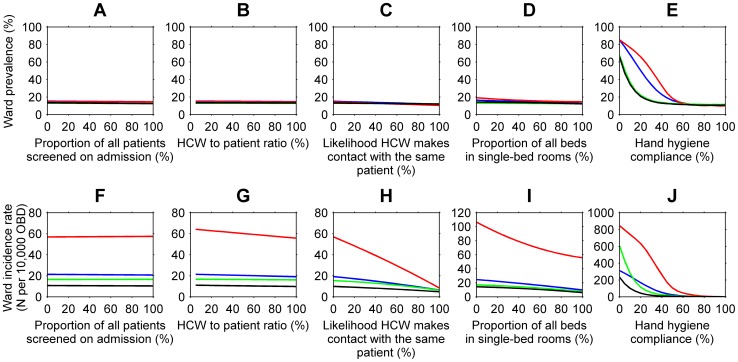
The effect of active surveillance (A and F), ward staffing levels or health care worker (HCW) to patient ratios (B and G), staff contact cohorting (C and H), patient isolation (D and I), and staff hand hygiene compliance (E and J) on the average daily prevalence and incidence rate per 10,000 overnight bed days (OBD) of MRSA, in surgical (blue), intensive care (red), short-stay medical (green), and long-stay medical (black) wards, in 2008 and 2009, averaged over 1000 simulations.

For all ward specialties, active surveillance for MRSA for reducing the average daily prevalence and incidence rate of MRSA appeared to be ineffective (Figure 3A, 3F). This finding differs from earlier studies [16], [38]. MRSA transmission was estimated to be higher in single-bed rooms than in multiple-bed rooms. Colonised patients detected through active surveillance and admitted to single-bed rooms, therefore, were modelled to have higher transmission rates than colonised patients in multiple-bed rooms. Despite this, the incidence rate of MRSA when active surveillance policies were in place did not exceed the baseline rate. This could be due to delays in the direct admission or placement of patients into single-bed rooms. Previous studies assumed no delay in the availability of MRSA screening test results and that patients could be cohorted immediately [38]–[40]. Like several authors [17], [41], we assumed a delay of two days for culture-based test results and same day results for PCR tests. Patient isolation in single-bed rooms was also delayed when single-bed rooms were not available. Active surveillance was limited by the isolation capacity, staff hand hygiene practice, staff contact cohorting and staffing levels of the hospital studied.

### Ward staffing levels

Despite additional staff, the average daily MRSA prevalence remained constant and few nosocomial MRSA cases were prevented throughout the hospital (Figure 3B, 3G). This result was constrained by the absence of staff contact cohorting and staff hand hygiene compliance levels kept consistent with current practice. This finding is supported by several studies [41]–[43]. In contrast, McBryde et al. [44] found that without staff contact cohorting, additional staff resulted in an increase in the incidence rate of MRSA. In the model by McBryde et al. [44] each health care worker made the same number of contacts daily; in our model, patients received the same number of contacts daily.

### Staff contact cohorting

The average daily prevalence and incidence rate of MRSA decreased with increased levels of staff contact cohorting (Figure 3C, 3H). The incidence rate was sensitive when greater than 85%, 70%, and 90% of all health care workers were cohorted in surgical, intensive care, and short-stay medical wards respectively. When health care workers were allocated to one patient each day, and did not break this cohort, the baseline MRSA incidence rate was reduced by 68%, 84%, 62%, and 53% in surgical, intensive care, short-stay medical, and long-stay medical wards respectively. The baseline average daily prevalence of MRSA decreased from 15% to 11% in surgical wards, 14% to 10% in intensive care wards and 13% to 12% in medical wards. Nosocomial MRSA colonisations could not be completely prevented as we assumed health care workers were cohorted with the same patient for one day only. Daily changes to the health care worker roster and patient turnover resulted in changes to the makeup of each cohort. Like Austin [42], we found staff contact cohorting to be more effective than increased staffing levels. Staff contact cohorting was found to rely heavily on adequate hand hygiene compliance and efficacy to be successful [45], [46].

### Patient isolation

The prevalence and incidence rate of MRSA decreased with, but were not sensitive to, additional single-bed rooms for patient isolation in surgical, intensive care, and short-stay medical wards (Figure 3D, 3I). In long-stay medical wards, large reductions in the incidence rate, relative to increases in isolation capacity, occurred when 85% or more of all beds were in single-bed rooms (|normalised sensitivity| > = 1). If all beds were in single-bed rooms, the baseline incidence rate in surgical and medical wards could be reduced by 38% - 48% in this setting. The baseline incidence rate in intensive care wards would double if all beds were in multiple-bed rooms. Previous studies found isolating patients colonised with MRSA in single-bed rooms to be more effective, but differ from our study in several assumptions [16], [38], [39]. First, previous studies assumed single-bed rooms were 100% efficacious; isolated patients did not contribute to further MRSA transmission. In our study, similar to Forrester and Pittet [47] and Raboud et al. [41], MRSA transmission could occur indirectly between patients in single-bed rooms. The probability of MRSA transmission upon contact was estimated to be higher in single-bed rooms than in multiple-bed rooms. For single-bed rooms that were additional to the baseline number of single-bed rooms, we assumed MRSA transmissibility to be 25% lower than that in multiple-bed rooms. MRSA transmissibility in beds in multiple-bed rooms that were additional to the baseline number of beds in multiple-bed rooms were assumed to be 25% higher that that in single-bed rooms. This explains the linear response in intensive care wards and non-linear response in surgical and medical wards to increased isolation capacity. Second, previous studies assumed isolation capacity was infinite or never reached. In this context, the number of single-bed rooms was fixed and, due to the high prevalence of MRSA, were often filled. We support the finding by Cooper et al. [39] that the effectiveness of patient isolation policies decreases as the capacity to isolate decreases.

### Staff hand hygiene compliance

**Figure 4:**
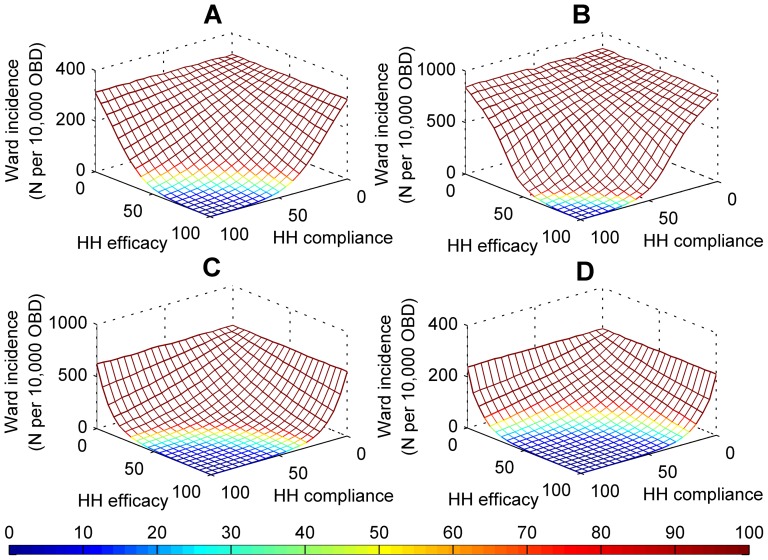
The incidence rate of MRSA per 10,000 overnight bed days (OBD), averaged over 1000 simulations, in response to changes in both staff hand hygiene (HH) compliance and hand hygiene efficacy in (A) surgical, (B) intensive care, (C) short-stay medical, and (D) long-stay medical wards. The colours indicate the MRSA incidence rate per 10,000 OBD. The dark blue area represents the combination of hand hygiene compliance and efficacy ranges that achieved incidence rates of less than 10 cases per 10,000 OBD.

**Figure 5:**
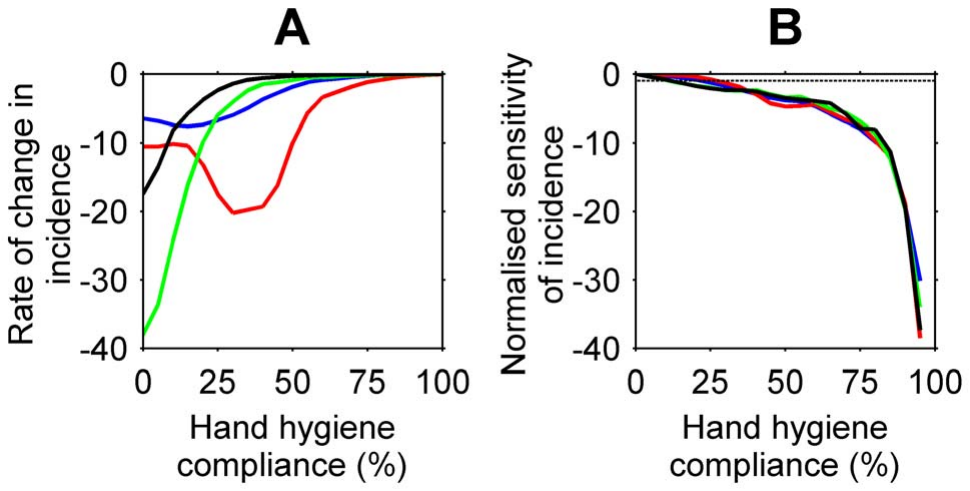
The change in the MRSA incidence rate per 10,000 overnight bed days (OBD) relative to changes in hand hygiene compliance levels. (A) The rate of change of the incidence rate per hand hygiene compliance level in surgical (blue), intensive care (red), short-stay medical (green), and long-stay medical (black) wards. (B) The normalised sensitivity of the incidence rate to increases in staff hand hygiene compliance for all ward specialties. The dotted line indicates when changes in the incidence rate are relatively larger than changes in the hand hygiene compliance level (|normalised sensitivity| > = 1).

Staff hand hygiene compliance appeared to be more successful in reducing the prevalence and incidence rate of MRSA than staff contact cohorting, patient isolation, additional staff, and active surveillance for MRSA. For all ward specialties, the average daily prevalence and incidence rate of MRSA decreased exponentially with increased staff hand hygiene compliance (Figure 3E, 3J). These findings are consistent with reports by Raboud et al. [41] and McBryde et al. [44]. The largest reductions in the average daily prevalence, relative to increases in staff hand hygiene compliance, occurred when compliance increased from 35% to 55% in surgical wards and 35% to 60% in intensive care wards (|normalised sensitivity| > = 1). Compliance levels achieved above the thresholds of 55% in surgical wards and 60% in intensive care wards resulted in small reductions in the average daily prevalence relative to reductions gained when compliance was lower. This finding is similar to previous studies [34], [44], [48]. Despite differences in ward characteristics, the decrease in ward prevalence was consistent across short- and long-stay medical wards. The incidence rates for all ward specialities were sensitive to increases in hand hygiene compliance from levels as low as 20% in some wards (30%, surgical wards; 35%, intensive care wards; 20%, medical wards). Nosocomial MRSA cases, acquired solely through the hands of health care workers, could be prevented in all wards with maximum hand hygiene compliance and efficacy. For this test, we assumed all MRSA pathogens were removed from the hands of heath care workers who performed hand hygiene, that is, hand hygiene efficacy was 100%. The efficacy of hand hygiene may differ across types of antiseptic and antimicrobial soaps and solutions [49]. The response of the incidence rate to the combination of staff hand hygiene compliance and hand hygiene efficacy is shown in Figure 4. The likelihood health care workers removed MRSA pathogens from their hands by performing hand hygiene was calculated as *hand hygiene compliance* x *hand hygiene efficacy*. Higher levels of hand hygiene compliance and efficacy were required in intensive care and surgical wards than in medical wards to reduce the incidence rate of MRSA. For example, for incidence rates of less than 10 cases per 10,000 OBD, with hand hygiene efficacy at 100%, hand hygiene compliance was found to be greater than 65%, 80%, 60%, and 40% in surgical, intensive care, short-stay medical, and long-stay medical wards respectively; with hand hygiene efficacy at 80%, hand hygiene compliance was found to be greater than 80%, 100%, 70%, and 50% in surgical, intensive care, short-stay medical, and long-stay medical wards respectively. The minimum prevalence level and incidence rate of MRSA in medical wards were reached at the same level of staff hand hygiene compliance. This minimising compliance level was lower than that for surgical and intensive care wards. This result, and the incidence rate in medical wards being sensitive from lower compliance levels than in surgical and intensive care wards (Figure 5), suggests there are fewer nosocomial cases in medical wards and that the prevalence of MRSA is composed primarily of existing MRSA cases rather than nosocomial MRSA cases. The greater number of nosocomial cases in surgical and intensive care wards could form a larger proportion of the MRSA prevalence in those wards.

### Limitations of the study

The transmission-related data, and parameter and model structure assumptions used in this study affect our results and conclusions. The data used to calibrate the model may underestimate the true MRSA prevalence and incidence rate in the hospital studied. Many MRSA colonised patients go undetected in wards where screening is limited. There may be insufficient epidemiological information to investigate potential nosocomial MRSA cases. The combination of the eight estimated MRSA transmission parameters used could be improved. Many values for each of the eight parameters and combinations of these values could produce a least squares fit between the simulated and observed MRSA prevalence and incidence rates in all ward specialties. A range of plausible values for these parameters could be sampled with care taken to sample suitable combinations of the eight transmission parameters. Optimising functions other than a least squares fit could also improve the parameter estimation process [50], [51]. The data collected was based on overnight census data. The results would differ greatly if the data collected reflected the entire patient throughput for a ward specialty in a single day [43]. We accounted for differences in transmission rates across room types and ward specialties. There may be population groups with different transmission rates, such as high risk patients, which have previously been modelled [16], [28]. Transmission parameters could be selected from a probability distribution function. A finer grained agent-based modelling approach could be applied so that the transmission probability reflects the characteristics and behaviours of each individual and organism strain [15], [28]. Our model could be extended to also incorporate MRSA transmission between (i) health care workers, (ii) health care workers or patients and the environment [47], and (iii) patients and visitors [15]. Several approaches have achieved this: an agent based approach, where different groups of people are defined [15], modelling the social network of individuals [52], or by adding an extra transmission event to reflect environmental transmission [47]. The availability of data for MRSA transmission from multiple sources is a limitation. Multiple strains of MRSA, each with different transmission rates, can circulate in a hospital, and can be modelled. Community-acquired MRSA (CA-MRSA), various strains of MRSA that have emerged in the community setting [53], has been modelled alongside hospital MRSA strains (HA-MRSA) in both the community and the hospital [40]. Pressley et al. [54] modelled co-colonisation with CA-MRSA and HA-MRSA. Austin and Anderson [55] calibrated their model for different strains of epidemic MRSA: EMRSA-15, EMRSA-16 and EMRSA-3, and vancomycin-resistant enterococci. An agent based approach can mark health care workers and patients as having multiple strains of MRSA and apply different transmission probabilities. Multiple transmission events could be modelled with transmission rates sampled from a distribution that reflects the diversity in the MRSA strain population.

## Conclusion

Hospital infection control interventions have differential impact on the prevalence and incidence rate of MRSA across ward specialties. These differences are likely to occur due to health care worker infection control behaviours and variations in the number of patients admitted to different wards with different capacities for isolation and contact precautions. Infection control policy targets should be specific for each ward specialty and the context of the hospital. With many policies dependent on each other, the effectiveness of infection control can be increased by applying carefully selected synergistic policies. Our study model can be calibrated to reflect different ward specialties and pathogens transmissible by contact and identify the most appropriate infection control policies and targets.
